# A *Picrorhiza kurroa* Derivative, Picroliv, Attenuates the Development of Dextran-Sulfate-Sodium-Induced Colitis in Mice

**DOI:** 10.1155/2012/751629

**Published:** 2012-10-16

**Authors:** De-Kui Zhang, Jian-Jie Yu, Yu-Min Li, Li-Na Wei, Yi Yu, Yan-Hu Feng, Xiang Wang

**Affiliations:** ^1^Department of Gastroenterology, The Second Hospital, Lanzhou University, Gansu Province, Lanzhou 730000, China; ^2^Key Laboratory of Digestive System Tumors, Gansu Province, Lanzhou 730000, China

## Abstract

*Background*. Free radicals and proinflammatory cytokines have been shown to play a critical role in the pathogenesis of ulcerative colitis (UC). Picroliv, a *Picrorhiza kurroa* derivative, has been demonstrated to have antioxidant and anti-inflammatory effect. The purpose of the study was to investigate the effects of picroliv on experimental model of UC in mice. *Materials and Methods*. Picroliv was administrated orally by gavage to mice with colitis induced by dextran sulfate sodium (DSS). Disease activity index (DAI), colon length, and histology score were observed. Myeloperoxidase (MPO) activity, and SOD, MDA concentrations were measured by enzyme-linked immunosorbent assay (ELISA) while the expression of cytokine mRNAs was studied by real-time-quantitative polymerase chain reaction and also ELISA. The expression of NF-**κ**B p65 was observed by immunohistochemistry staining and western blotting. *Results*. A significant improvement was observed in DAI and histological score in mice treated with picroliv, and incerased MPO activity, MDA concentrations, and the expression of IL-1**β**, TNF-**α**, and NF-**κ**B p65 in mice with DSS-induced colitis were significantly reduced while decreased SOD level increased following administration of picroliv. *Conclusion*. The administration of picroliv leads to an amelioration of DSS-induced colitis, suggesting administration of picroliv may provide a therapeutic approach for UC.

## 1. Introduction

Ulcerative colitis (UC) is a chronic inflammatory bowel disease of unknown cause that exhibits an unpredictable clinical course with remissions and exacerbation and is characterized by rectal bleeding and diarrhea [1]. Although great advances have been made in the management of the disease, no definitive therapies until now are available for this disorder because the exact pathogenesis is elusive [2, 3]. Conventional treatments for UC include aminosalicylates and corticosteroids as mainstays of therapy. Immunosuppressive agents, such as azathioprine, 6-mercaptopurine, and methotrexate, are used for corticosteroid-resistant or -dependent patients. However, these drugs are not always effective and may inflict serious side effects [2–4]. Recently, a biologic agent, antitumor necrosis factor alpha (anti-TNF-*α*) antibody (infliximab), has been shown to be effective in clinical application; however, infliximab can cause serious adverse reactions such as increased risk of infection, hypersensitivity, and anti-antibody reaction and an unknown risk of mutagenesis [5, 6]. Therefore, new therapeutic strategies are awaited.

 Although the precise mechanism of UC still remains unknown, there are accumulating evidences that the increase of proinflammatory cytokines such as IL-1*β* and TNF-*α* within colonic tissues plays a pivotal role in the pathogenesis of UC [7–9]. Therefore, blockade of these inflammatory mediators or their upstream regulators such as NF-*κ*B p65 can offer an alternative therapy for UC [5, 6, 10, 11]. Also, many studies have showed that reactive oxygen species (ROS) are increased in inflammatory bowel disease (IBD) and overproduction of colonic oxidants contributes to mucosal injury in IBD [12, 13], thus some agents exert protective effects on IBD by antioxidant mechanism [14, 15]. 

 Recent studies have unequivocally shown that the expressions of TNF-*α* and IL-1*β* are regulated by the transcription factor nuclear factor kappa B(NF-*κ*B); NF-*κ*B plays a central role in immune and inflammatory responses and may be a good target for therapy [16, 17]. NF-*κ*B is mostly composed of RelA (p65) and NF-*κ*B1(p50); these NF-*κ*B dimers are kept in an inactive cytoplasmic complex by inhibitory proteins, the inhibitor protein kappa B(I*κ*B) family, in resting cells. NF-*κ*B can be activated within minutes by a variety of stimuli, including inflammatory molecules such as TNF-*α* and IL-1*β*, growth factors, bacterial lipopolysaccharide (LPS), and oxidative stress, which induce site-specific phosphorylation of I*κ*B and consecutive rapid dissociation of the complex accompanied by proteolytic degradation of I*κ*B. The released NF-*κ*B proteins subsequently transmigrate from cytoplasm into the nucleus where they can induce gene transcription by binding to specific promoter elements [18–20]. Activated NF-*κ*B has been demonstrated in colonic epithelial cells and macrophages of patients with IBD [21, 22], and also NF-*κ*B p65 antisense oligonucleotide treatment was reported to have much benefits in experimental colitis, although toxicity effects must be carefully analyzed [23, 24]. The above data predict that NF-*κ*B may be a new and more effective therapy target in experimental colitis.

 Picroliv is a mixture of two iridoid glycosides, Picroside-1 and Kutkoside (1.0 : 1.5, w/w), purified from the roots and rhizomes of the plant *Picrorhiza kurroa*, a perennial herb popularly known as “Kutki” or “Kurro” which is used to treat a variety of ailments, including fever, hepatitis, allergies, asthma, and other inflammatory diseases [25, 26]. Studies have shown that picroliv exhibits hepatoprotective effect against aflatoxin [27–29], oxytetracycline [30], carbon tetrachloride [31, 32], paracetamol [33], and alcohol [34]; protects against ischemia reperfusion injury of the liver [35] and kidneys [36]; and exhibits anti-inflammatory [37], immunomodulatory [38, 39] and anticarcinogenic [40–43] effects. Studies have revealed that picroliv mediates these effects by changing the antioxidant status of cells [44], downregulating the expressions of c-Jun and c-fos [45], and inhibiting hypoxia-induced downregulation of insulin-like growth factor-1 and -2 [46]. Because prooxidant, proinflammatory, immunomodulatory, and carcinogenic effects have been linked with activation of NF-*κ*B, furthermore recent studies shown that picroliv can inhibit NF-*κ*B activation [47]. These results are of great interest and, based on this finding, it could be speculated that picroliv is useful in the treatment of patients with UC. Unfortunately, to date, there has been no information on whether picroliv is therapeutic for UC.

 In the present study we investigated the protective effect of picroliv on a well-defined murine model of dextran-sulfate-sodium- (DSS-) induced colitis, which resembles human UC, aimed to provide experimental evidences that picroliv may serve as a possible treatment for human with UC.

## 2. Materials and Methods

### 2.1. Animals

Seven-week-old female BALB/c mice weighing about 18–22 g were obtained from the Experimental Animal Center of Sichuan University, Chengdu, China, and housed in cages at room temperature 25°C with alternating 12:12 h light–dark cycles. Standard mouse chow pellets and water were supplied ad libitum. This study was approved by the Animal Ethics Committee of West China Hospital, Sichuan University.

### 2.2. Induction of Colitis and Administration of Picroliv

DSS (molecular mass, 36,000–50,000 Da) was obtained from ICN Biomedicals (Ohio, USA) and dissolved in distilled water. Colitis was induced by drinking distilled water ad libitum containing 5% DSS (w/v) from day 0 for 7 days. The mice were randomly divided into three groups, the normal control group (control group, *n* = 10) received distilled water for 14 days; the saline control group (DSS group, *n* = 10) received 5% DSS for 7 days and 0.5 mL of saline given orally by gavage from day 8 then continued for an additional 7 days. Picroliv treatment group (picro group, *n* = 10) received 5% DSS for 7 days and 12.5 mg/kg per day of picroliv orally by gavage from day 8 then continued for an additional 7 days. Picroliv were purchased from Central Drug Research Institute, Lucknow, India, and dissolved in dimethyl-sulphoxide (DMSO), diluted in 0.9% saline solution. The total amount of DMSO did not exceed 1% upon testing, an amount which was considered of no significance in the assays used. Dosage was selected based on the previous *in vivo* studies and was confirmed to result in effective anti-inflammatory activity [26, 48]. In this model, mice were checked daily for behaviour, body weight, stool blood, and stool consistency.

### 2.3. Assessment of DSS-Induced Colitis

A disease activity index (DAI) was determined by scoring changes in body weight, stool hemoccult positivity or gross bleeding, and stool consistency in accordance with the method described by Murthy et al. [49] (Table 1). Colon length (cm) as an indirect marker of inflammation was observed also.

### 2.4. Histologic Assessment of Colon Damage

After mice were killed under anesthesia, their colons were immediately removed and fixed in 10% buffered formalin, paraffin-embedded, sectioned, and stained with hematoxylin and eosin (H&E). Histological score of H&E-stained specimens of the colon was determined by two pathologists in a blinded fashion according to the method reported by Ten Hove et al. [50]. The mean score in each section was calculated.

### 2.5. Tissue Myeloperoxidase Activity

The colon samples were washed with cold PBS, blotted dry, and were immediately thawed for the myeloperoxidase activity according to manufacturer's instructions (CytoStore, Alberta, Canada).

### 2.6. Real-Time-Quantitative Polymerase Chain Reaction (RT-PCR) for Expression of Cytokine mRNAs in Colon Tissues

 Total RNA was isolated from colonic tissues using RNAqueous (Ambion, Austin, TX, USA). The amount of RNA was estimated by measuring the absorbance at 260 nm. The Applied Biosystems (Foster City, CA, USA) assays-by-design or assays-on-demand 20 × assay mix of primers and TaqMan MGB probes (FAM dye-labeled) were used for all of the target genes and predeveloped 18S rRNA (VIC-dye-labeled probe). TaqMan assay reagent (P/N 4319413E) was used for endogenous control. These assays are designed to span exon–exon junctions so as not to detect genomic DNA, and all these primers and probe sequences were searched against the Celera database to confirm specificity. The primer and probe sequences used were as follows: IL-1*β*: probe: 5-CCATCAGAGGCAAGGAGGAA; primer: sense: 5′-TCGCTCAGGGTCACAAAGAAA, antisense: 5′-CCATCAGAGGCAAGGAGGAA; TNF-*α*: probe: 5′-CCCGACTACGTGCTCCTCACCCA, primer: sense: 5′-TCTCTTCAAGGGACAAGGCTG, antisense: 5′-ATAGCAAATCGGCTGACGGT. Separate tubes (singleplex) one-step reverse transcription (RT)-polymerase chain reaction (PCR) was performed with 80 ng RNA for both target genes and endogenous control. The reagent we used was TaqMan one-step RT-PCR master mix reagent kit (P/N 4309169). The cycling parameters for one-step RT-PCR were reverse transcription 48°C for 30 min, AmpliTaq activation 95°C for 10 min, denaturation 95°C for 15 s, and annealing-extension 60°C for 1 min (repeated 40 times) on ABI7000 (Applied Biosystems, Foster City, CA, USA). Duplicate CT values were analyzed in Microsoft Excel using the comparative CT(^ΔΔ^C_T_) method as described by the manufacturer. The amount of target (2^-ΔΔCT^) was obtained by normalizing it to an endogenous reference (18S rRNA) and relative to a calibrator.

### 2.7. Enzyme-Linked Immunosorbent Assay

The colon tissues were rinsed and weighed, then put into tubes with 9 volumes of 9 g/L normal saline. Then homogenized for 10 minutes and centrifugated at 4000 r/min for 10 minutes at 4°C. The concentrations of IL-1*β* and TNF-*α* in homogenized colon tissues were measured by enzyme-linked immunosorbent assay (ELISA) kits according to the manufacturer's instructions (R&D Systems, Minneapolis, MN).

### 2.8. Assessment of SOD, MDA Level

The colon tissues were rinsed and weighed, then put into tubes with 9 volumes of 9 g/L normal saline. Then the tissue samples were homogenized for 10 minutes. After centrifugation at 4000 r/min for 10 minutes at 4°C, the MDA contents and SOD activities in the supernatant were measured by the assay kit (Nanjing Jincheng Corp, China) according to its provider's instructions.

### 2.9. Immunohistochemistry Staining for NF-*κ*B

Sections of colon tissues were deparaffinized in xylene and hydrated in a series of graded alcohol. After dewaxing and rehydration, the antigen retrieval was done by microwave for 15 minutes. Sections were immersed in 3% hydrogen peroxide in methanol for 20 minutes at room temperature to abolish endogenous peroxidase activities and then they were blocked with normal goat serum at 37°C for 15 minutes. Slides were incubated with polyclonal antibody of NF-*κ*B (diluted to 1 : 200, Santa Cruz Biotechnology) at 37°C for 60 minutes. After PBS washing, the slides were incubated with a biotinylated horse peroxidase-conjugated secondary antibody and 0.1% DAB substrate, using the standard streptavidin-biotin-based method. Incubation with PBS instead of the primary antibody served as a negative control. The positive cells were observed and evaluated by two independent observers. A cytoplasmic or nuclear brown granule was marked as a positive expression of NF-*κ*B. The results were evaluated semiquantitatively according to the percentage of positive cells in ten randomly selected fields under high-power microscope (400-fold magnification) for each sample.

### 2.10. Western Blotting for NF-*κ*B

Nuclear proteins were extracted by the method of manufacturer's instructions (EMD Millipore Corporation, Billerica, MA, USA). To determine the levels of NF-*κ*B protein expression in the nucleus, extracts were fractionated using sodium dodecyl sulfate (SDS)-polyacrylamide gel electrophoresis (PAGE) as described previously and analyzed by western blotting according to the standard protocols [51]. The proteins were then electrotransferred to nitrocellulose membranes and blotted with NF-*κ*B p65 antibody (1 : 400), anti-*β*-actin antibody (1 : 500). All of the antibodies were purchased from Santa Cruz Biotechnology (Santa Cruz, CA, USA).

### 2.11. Data Analysis

Data were expressed as means ± SEM. Statistical analysis was performed with SPSS 13.0 statistical software. The Student's *t*-test or analysis of variance was used for data analysis. A value of *P* < 0.05 was considered statistically significant.

## 3. Results

### 3.1. Effects of Picroliv on Clinical Indices and Histological Injury Scores

**Figure 1:**
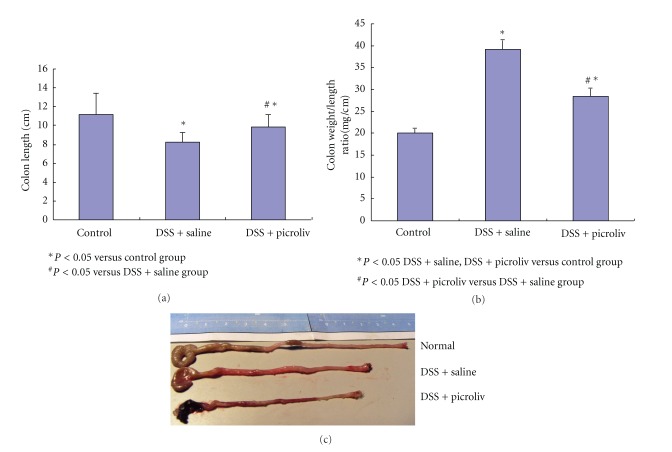
(a) The effect of picroliv on colon length. (b) The effect of picroliv on colon weight/length ratio. (c) Macroscopic changes of the colons.

**Figure 2:**
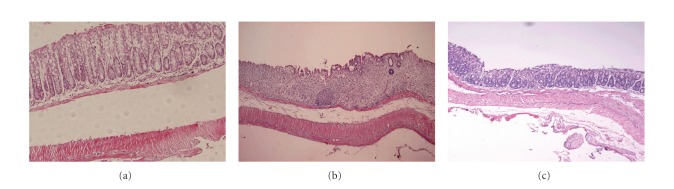
Effect of picroliv on histological injury in DSS-induced colitis. Hematoxylin and eosin staining (magnification ×100) of colonic tissue section. (a) Mucosa from control mice did not show any histological modifications; (b) DSS-induced mucosal injury associated with complete destruction of epithelial architecture with loss of crypts and epithelial integrity, submucosal edema, and intense inflammatory cellular infiltration; (c) treatment with picroliv attenuated the disturbances in morphology but caused a mild cellular infiltration.

Treatment of BALB/c mice with 5% DSS in their drinking distilled water for 7 days resulted in clinical, gross, and histological signs of colitis. Mice produced loose stool or diarrhoea, occult or gross rectal bleeding, and weight loss. Treatment with picroliv significantly reduced the clinical disease activity index (DAI) of colitis with the dose of 12.5 mg/kg per day for 7 days compared with DSS-treated mice (score 1.35 ± 0.26 versus 3.26 ± 0.32, *P* < 0.05) (Table 2). In DSS group, the severe colitis caused by DSS was associated with a significant (*P* < 0.05) shortening of the colon length compared with healthy controls (normal group). However, mice in the picroliv-treated group had significantly longer colons than the saline group although it is length was shorter than normal group, statistically significant (Figure 1). Consistent with the clinical features, treatment of DSS-fed mice with picroliv showed a significant improvement in histological injury scores compared with the saline-treated group (*P* < 0.05) (Table 2, Figure 2).

### 3.2. Effects of Picroliv on MPO Activity

MPO is an enzyme found in neutrophils and its activity in the colon is linearly related to neutrophil infiltration. Evaluation of leukocyte recruitment was assessed by the measurement of MPO activity. As shown in Table 3, MPO activity levels were low in the colonic tissues of normal control mice and markedly increased in mice with DSS-induced colitis. The increased MPO activity in mice with DSS-induced colitis was significantly reduced after administration of picroliv.

### 3.3. Effects of Picroliv on SOD, MDA Concentration

As shown in Table 3, compared with the normal control group, the activities of SOD notably decreased and the contents of MDA significantly increased in saline-treated mice with DSS-induced colitis (*P* < 0.05). Administration with picroliv could elevate the activities of SOD and reduce the contents of MDA (*P* < 0.05).

### 3.4. Effects of Picroliv on Key Inflammatory Cytokine Expression

As shown in Table 4, a significant increase of mRNA and protein expression of IL-1*β* and TNF-*α*, as assessed by real-time quantitative RT-PCR and ELISA, respectively, was observed in saline-treated mice with DSS-induced colitis compared with the normal control group (*P* < 0.05). The increase in the amount of IL-1*β* and TNF-*α* mRNA and protein in the mice with DSS-induced colitis was reduced significantly following the treatment with picroliv.

### 3.5. Effects of Picroliv on NF-*κ*B p65 Expression

**Figure 3:**
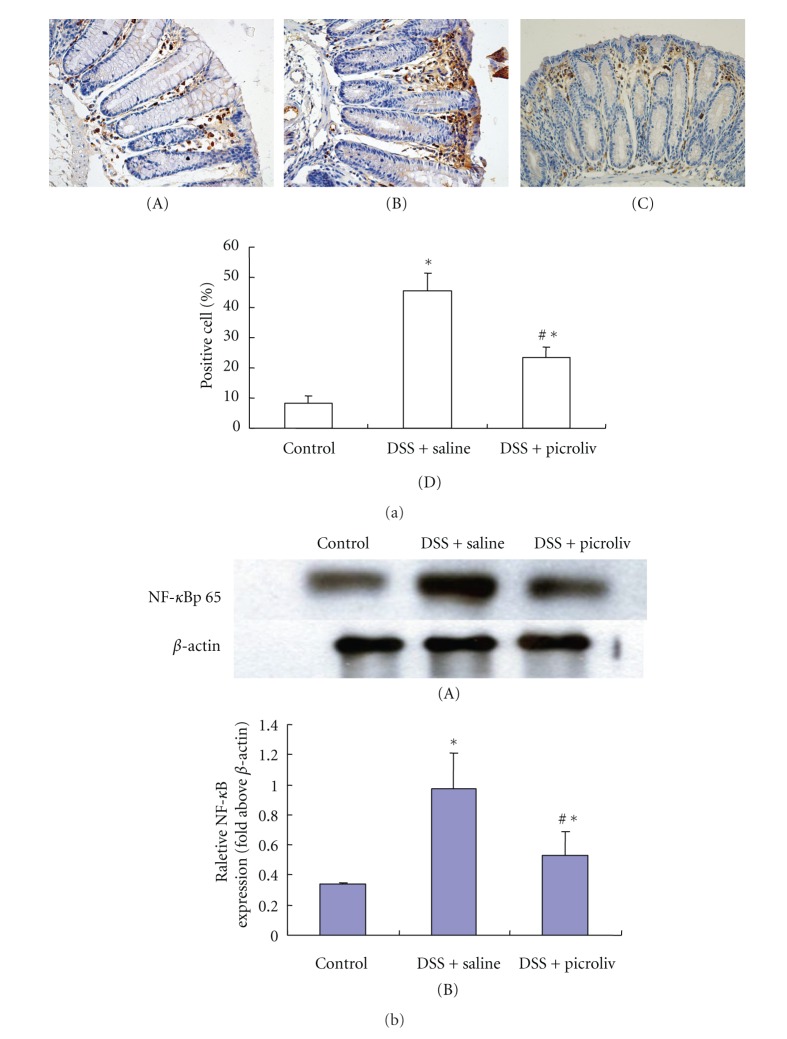
(a) Effect of picroliv on NF-*κ*B p65 expression. Immunohistochemical staining for NF-*κ*B p65 expression in (magnification ×200), (A) Control group, (B) DSS+saline group, (C) picroliv group, (D) positive cell percent of the expression of NF-*κ*B p65. The figure shows that DSS treatment significantly increased the expression of NF-*κ*B p65, which was decreased significantly with picroliv. Data are expressed as mean ± SE (each group, *n* = 10). ^*∗*^ *P* < 0.05 DSS + saline, DSS + picroliv versus control group, ^#^ *P* < 0.05 DSS + picroliv versus DSS + saline group. (b) Effects of picroliv on NF-*κ*B p65 expression. (A) Western-blot results display protein expression of NF-*κ*B p65 in the colonic tissues. (B) Densitometric analysis of the expression of NF-*κ*B p65. DSS treatment significantly increased the expression of NF-*κ*B p65, which was decreased significantly with picroliv. Data are expressed as mean ± SE (each group, *n* = 10). ^*∗*^ *P* < 0.05 DSS + saline, DSS + picroliv versus control group, ^#^ *P* < 0.05 DSS + picroliv versus DSS + saline group.

As shown in Figure 3, the expression of NF-*κ*B p65, as assessed by immunohistochemistry staining and western blotting, was markedly increased in saline-treated mice with DSS-induced colitis compared with normal control group (*P* < 0.05). Treatment with picroliv resulted in a significant reduction of NF-*κ*B p65 protein expression in mice with DSS-induced colitis in comparison with saline-treated control group (*P* < 0.05).

## 4. Discussion

The use of natural anti-inflammatory products provides an attractive and relatively nontoxic alternative to modulate inflammatory disorders. Picroliv, A *Picrorhiza kurroa* derivative, has been demonstrated to have antioxidant, anti-inflammatory effect and inhibit the expression of NF-*κ*B [37, 44, 47]. However, there is still lack of information whether picroliv can attenuate intestinal inflammatory disease. In the present study we administered picroliv orally after DSS consumption (postadministration) in mice, then measured the severity of colitis by assessing the body weight loss, stool consistency, and stool blood, and evaluated the therapeutic effects of picroliv treatment. Our findings demonstrate that picroliv treatment significantly suppressed DSS-induced colitis in mice by improving their body weight and stool consistency as well as decreasing intestinal bleeding. In addition, the DSS-induced colitis exhibited mucosal inflammation with extensive infiltration of leukocytes and excessive production of reactive oxygen species (ROS) in the mucosa. Picroliv treatment greatly reduced the infiltration of leukocytes and mucosal damage via the downregulation of MPO activity and MDA level while upregulating SOD activity, resulting in significant amelioration of histopathology scores and preserving colon length. Meanwhile oral administration of picroliv significantly reduced IL-1*β*, TNF-a mRNA expression increased in the clonic tissues of DSS-induced colitis by inhibiting the expression of NF-*κ*B p65. 

Overexpression of inflammatory cytokines, especially IL-1*β* and TNF-*α*, is thought to play an important role in the pathogenesis of UC [7, 8]. These proinflammatory cytokines amplify the inflammatory cascade of inflammatory mediators, destructive enzymes, and free radicals that cause tissue damage [52]. Therefore, blockade of these cytokines can offer an alternative therapy for UC. Indeed, several biologic agents that block the actions of IL-1*β* and TNF-*α* have been successfully used in experimental colitis models [9, 53]. Moreover, anti-TNF antibody such as infliximab has been shown to be effective in human trials [54, 55]. In the present study, we observed an elevation of the proinflammatory cytokines, such as TNF-*α* and IL-1*β*, in DSS colitis tissue. However, oral administration of picroliv greatly inhibited the transcripts of these cytokines. The present results indicated that picroliv may be a choice to treatment for DSS-induced colitis.

However, it is well known that, in addition to the increase of IL-1*β* and TNF-*α*, other proinflammatory cytokines and inflammatory mediators such as IL-6, IL-8, intercellular adhesion molecule-1 (ICAM-1), matrix metalloproteinase, COX-2, and iNOS have also been demonstrated to be increased in UC. All these inflammatory mediators are considered to play a vital role in inflammatory process of UC [7, 8]. Therefore, it is insufficient for achieving the maximum therapeutic effects only to block individual factors in a multifactorial disease such as UC. Actually, individual factors such as cytokines or adhesion molecules only represent a downstream target, whereas NF-*κ*B is just the final common pathway or rate-limiting step in the inflammatory cascade. Obviously, as a therapeutic target, NF-*κ*B holds great promise. Therefore, in order to achieve the maximum therapeutic effects, using a therapeutic approach that interferes with a central player (upstream target) such as NF-*κ*B in the cascade of inflammation, namely, blocking simultaneously the expression of multiple inflammatory mediators, may be more effective than blocking individual factors such as TNF-*α*, IL-1*β* in a multifactorial inflammatory disease [56]. Indeed in our study activated NF-*κ*B has been found in intestinal mucosa from DSS-induced colitis and overexpressed NF-*κ*B proteins extracted from nucleus were significantly inhibited after administration orally of picroliv. This result is consistent with previous data indicating that picroliv inhibited activation of I*κ*B*α* kinase, leading to inhibition of phosphorylation and degradation of I*κ*B*α*. It also inhibited phosphorylation and nuclear translocation of p65. Further studies revealed that picroliv directly inhibits the binding of NF-*κ*B p65 to DNA [47]. Therefore, we believe that picroliv may be an efficacious and promising remedy in the treatment for UC.

 Oxidative stress is believed to be a key factor in the pathogenesis and perpetuation of the mucosal damage in IBD. Accumulation of ROS in ulcerative colon tissues stimulates inflammation responses and secretion of proinflammatory cytokines, such as TNF-*α*, IL-1 and IL-6 [57, 58]. ROS also impairs the integrity of the intestinal epithelial cells and increases the intestinal mucosal permeability, which subsequently attenuates the barrier function and host defense to exogenous bacteria and microorganisms [59, 60]. In addition, ROS could induce DNA damage and stimulate activation of NF-*κ*B that plays an important role in inflammation responses [58, 61]. MDA is a product of polyunsaturated fatty acids oxidated and frequently used in the measurement of lipid peroxide levels. Elevated levels of MDA are shown in IBD also. Two cytoplasmic enzymes, superoxide dismutase (SOD) and myeloperoxidase (MPO), protect the cell contents against oxidizing activity by destroying superoxide anions (–O_2_) and hydrogen peroxide (H_2_O_2_), respectively [62]. At present, many therapies based on SOD have been applied in treatment of IBD. Suzuki et al. [63] reported that PC-SOD (40 mg or 80 mg daily) is able to improve UC rapidly. Also MPO is one of the indicators of inflammation and it is well correlated with neutrophil infiltration in various colitis models. SOD reduces the oxidative stress and the activation of mediators of inflammatory response [64]. However, our study found that increased MDA level and MPO activity and reduced SOD activity in colonic tissue of DSS-colitis group were significantly improved after oral administration of picroliv accompanying with the amelioration of inflammation.

In conclusion, this study demonstrates that the degree of colitis caused by administration of DSS is significantly attenuated by picroliv. The anti-inflammatory effects of picroliv are associated with a reduction in the upregulation of proinflammatory cytokines IL-1*β* and TNF-*α* through suppression of NF-*κ*B, attenuation of the recruitment of neutrophil, and releasing of lipid peroxidation. Being a relatively nontoxic natural product, combined with its excellent anti-inflammatory activity, immunomodulatory [39], and anticarcinogenic [40–43] effects, picroliv could supply a good choice to the treatment for IBD and need be studied further.

## Figures and Tables

**Table 1:** Scoring of disease activity index.

Score	Weight loss (%)	Stool consistency	Occult-gross bleeding
0	None	Normal	Normal
1	1–5		
2	5–10	Loose stools	Guaiac (+)
3	10–15		
4	>20	diarrhea	Gross bleeding

DAI value is the combined scores of weight loss, stool consistency, and bleeding divided by 3.

**Table 2:** The effect of picroliv on clinical indices and histological injury scores.

Group	*N *	DAI	Histological scores
Control	10	0	0
DSS + saline	10	3.26 ± 0.32	11.5 ± 3.25
DSS + picroliv	10	1.35 ± 0.26*	5.6 ± 1.21*

Results are expressed as the mean ± SEM.

^*∗*^ *P* < 0.05 versus control group, DSS + saline group.

**Table 3:** Effect of picroliv on MPO activity and the levels of SOD and MDA of colon tissues in mice.

Group	*N*	SOD IU/mg protein	MDA nmol/mg	MPO IU/mg protein
Control	10	50.23 ± 3.58	2.21 ± 0.03	5.94 ± 1.23
DSS + saline	10	24.25 ± 2.66	5.87 ± 1.33	35.35 ± 4.68
DSS + picroliv	10	36.49 ± 2.18*	3.89 ± 1.42*	16.01 ± 3.34*

Results are expressed as the mean ± SEM.

^*∗*^ *P* < 0.05 versus control group, DSS + saline group.

**Table 4:** The effect of picroliv on IL-1*β* and TNF-*α* genes and proteins expression.

Group	*N*	IL-1*β*		TNF-*α*	
		mRNA	Pg/mg protein	mRNA	Pg/mg protein
Control	10	0.026 ± 0.004	100.26 ± 3.84	0.034 ± 0.002	50.27 ± 8.51
DSS + saline	10	0.686 ± 0.121	2338.68 ± 210.12	0.874 ± 0.231	486.44 ± 54.31
DSS + Picroliv	10	0.253 ± 0.088*	856.25 ± 32.15*	0.331 ± 0.147*	310.66 ± 22.38*

Results are expressed as the mean ± SEM.

^*∗*^ *P* < 0.05 versus control group, DSS + saline group.

## References

[1] Fiocchi C (1998). Inflammatory bowel disease: etiology and pathogenesis. *Gastroenterology*.

[2] Podolsky DK (2002). Inflammatory bowel disease. *New England Journal of Medicine*.

[3] Sands BE (2000). Therapy of inflammatory bowel disease. *Gastroenterology*.

[4] Sandborn WJ, Targan SR (2002). Biologic therapy of inflammatory bowel disease. *Gastroenterology*.

[5] Ardizzone S, Porro GB (2005). Biologic therapy for inflammatory bowel disease. *Drugs*.

[6] Reddy JG, Loftus EV (2006). Safety of infliximab and other biologic agents in the inflammatory bowel diseases. *Gastroenterology Clinics of North America*.

[7] Papadakis KA, Targan SR (2000). Role of cytokines in the pathogenesis of inflammatory bowel disease. *Annual Review of Medicine*.

[8] Rogler G, Andus T (1998). Cytokines in inflammatory bowel disease. *World Journal of Surgery*.

[9] Ogata H, Hibi T (2003). Cytokine and anti-cytokine therapies for inflammatory bowel disease. *Current Pharmaceutical Design*.

[10] Nakamura K, Honda K, Mizutani T, Akiho H, Harada N (2006). Novel strategies for the treatment of inflammatory bowel disease: selective inhibition of cytokines and adhesion molecules. *World Journal of Gastroenterology*.

[11] Xiang JY, Wu LG, Huang XL (2009). Amelioration of murine dextran sulfate sodium-induced colitis by nuclear factor-*κ*b decoy oligonucleotides. *American Journal of Surgery*.

[12] Najafzadeh M, Reynolds PD, Baumgartner A, Jerwood D, Anderson D (2007). Chaga mushroom extract inhibits oxidative DNA damage in lymphocytes of patients with inflammatory bowel disease. *BioFactors*.

[13] Roessner A, Kuester D, Malfertheiner P, Schneider-Stock R (2008). Oxidative stress in ulcerative colitis-associated carcinogenesis. *Pathology Research and Practice*.

[14] Dost T, Ozkayran H, Gokalp F, Yenisey C, Birincioglu M (2009). The effect of *Hypericum perforatum* (St. John’s Wort) on experimental colitis in rat. *Digestive Diseases and Sciences*.

[15] Te Velde AA, Pronk I, De Kort F, Stokkers PCF (2008). Glutathione peroxidase 2 and aquaporin 8 as new markers for colonic inflammation in experimental colitis and inflammatory bowel diseases: an important role for H_2_O_2_?. *European Journal of Gastroenterology and Hepatology*.

[16] Baldwin AS (2001). Series introduction: the transcription factor NF-*κ*B and human disease. *Journal of Clinical Investigation*.

[17] Jobin C, Balfour Sartor R (2000). NF-*κ*B signaling proteins as therapeutic targets for inflammatory bowel diseases. *Inflammatory Bowel Diseases*.

[18] Glasgow JN, Wood T, Perez-Polo JR (2000). Identification and characterization of nuclear factor *κ*B binding sites in the murine bcl-x promoter. *Journal of Neurochemistry*.

[19] Tak PP, Firestein GS (2001). NF-*κ*B: a key role in inflammatory diseases. *Journal of Clinical Investigation*.

[20] Ghosh S, May MJ, Kopp EB (1998). NF-*κ*B and rel proteins: evolutionarily conserved mediators of immune responses. *Annual Review of Immunology*.

[21] Schreiber S, Nikolaus S, Hampe J (1998). Activation of nuclear factor *κ*B inflammatory bowel disease. *Gut*.

[22] Rogler G, Brand K, Vogl D (1998). Nuclear factor *κ*B is activated in macrophages and epithelial cells of inflamed intestinal mucosa. *Gastroenterology*.

[23] Neurath MF, Pettersson S, Meyer Zum Buschenfelde KH, Strober W (1996). Local administration of antisense phosphorothioate oligonucleotides to the p65 subunit of NF-*κ*B abrogates established experimental colitis in mice. *Nature Medicine*.

[24] Neurath MF, Pettersson S (1997). Predominant role of NF-*κ*B p65 in the pathogenesis of chronic intestinal inflammation. *Immunobiology*.

[25] Mehrotra R, Rawat S, Kulshreshtha DK, Patnaik GK, Dhawan BN (1990). In vitro studies on the effect of certain natural products against hepatitis B virus. *Indian Journal of Medical Research Section B*.

[26] Baruah CC, Gupta PP, Nath A, Patnaik LGK, Dhawan BN (1998). Anti-allergic and anti-anaphylactic activity of picroliv—a standardised iridoid glycoside fraction of *Picrorhiza kurroa*. *Pharmacological Research*.

[27] Rastogi R, Srivastava AK, Srivastava M, Rastogi AK (2000). Hepatocurative effect of picroliv and silymarin against aflatoxin B1 induced hepatotoxicity in rats. *Planta Medica*.

[28] Rastogi R, Srivastava AK, Rastogi AK (2001). Long term effect of aflatoxin B1 on lipid peroxidation in rat liver and kidney: effect of picroliv and silymarin. *Phytotherapy Research*.

[29] Rastogi R, Srivastava AK, Rastogi AK (2001). Biochemical changes induced in liver and serum of aflatoxin B1-treated male Wistar rats: preventive effect of Picroliv. *Pharmacology and Toxicology*.

[30] Saraswat B, Visen PKS, Patnaik GK, Dhawan BN (1997). Protective effect of picroliv, active constituent of *Picrorhiza kurrooa*, against oxytetracycline induced hepatic damage. *Indian Journal of Experimental Biology*.

[31] Santra A, Das S, Maity A, Rao SB, Mazumder DNG (1998). Prevention of carbon tetrachloride-induced hepatic injury in mice by *Picrorhiza kurrooa*. *Indian Journal of Gastroenterology*.

[32] Girish C, Pradhan SC (2012). Hepatoprotective activities of picroliv, curcumin, and ellagic acid compared to silymarin on carbon-tetrachloride-induced liver toxicity in mice. *Journal of Pharmacology and Pharmacotherapeutics*.

[33] Girish C, Koner BC, Jayanthi S, Ramachandra Rao K, Rajesh B, Pradhan SC (2009). Hepatoprotective activity of picroliv, curcumin and ellagic acid compared to silymarin on paracetamol induced liver toxicity in mice. *Fundamental and Clinical Pharmacology*.

[34] Rastogi R, Saksena S, Garg NK, Kapoor NK, Agarwal DP, Dhawan BN (1996). Picroliv protects against alcohol-induced chronic hepatotoxicity in rats. *Planta Medica*.

[35] Singh AK, Mani H, Seth P (2000). Picroliv preconditioning protects the rat liver against ischemia-reperfusion injury. *European Journal of Pharmacology*.

[36] Seth P, Kumari R, Madhavan S (2000). Prevention of renal ischemia-reperfusion-induced injury in rats by picroliv. *Biochemical Pharmacology*.

[37] Jia Q, Hong MF, Minter D (1999). Pikuroside: a novel iridoid from *Picrorhiza kurroa*. *Journal of Natural Products*.

[38] Sinha S, Mehrotra J, Bala L, Jaiswal AK, Dhawan BN (1998). Picroliv, the iridoid glycoside fraction of *Picrorhiza kurroa*, selectively augments human T cell response to mycobacterial protein antigens. *Immunopharmacology and Immunotoxicology*.

[39] Shakya N, Sane SA, Gupta S (2011). Antileishmanial efficacy of fluconazole and miltefosine in combination with an immunomodulator—picroliv. *Parasitology Research*.

[40] Jeena KJ, Joy KL, Kuttan R (1999). Effect of *Emblica officinalis, Phyllanthus amarus* and *Picrorrhiza kurroa* on N-nitrosodiethylamine induced hepatocarcinogenesis. *Cancer Letters*.

[41] Rajeshkumar NV, Kuttan R (2000). Inhibition of N-nitrosodiethylamine—induced hepatocarcinogenesis by Picroliv. *Journal of Experimental and Clinical Cancer Research*.

[42] Rajeshkumar NV, Kuttan R (2001). Protective effect of Picroliv, the active constituent of *Picrorhiza kurroa*, against chemical carcinogenesis in mice. *Teratogenesis Carcinogenesis and Mutagenesis*.

[43] Rajeshkumar NV, Kuttan R (2003). Modulation of carcinogenic response and antioxidant enzymes of rats administered with 1,2-dimethylhydrazine by Picroliv. *Cancer Letters*.

[44] Chander R, Kapoor NK, Dhawan BN (1992). Picroliv, picroside-I and kutkoside from *Picrorhiza kurrooa* are scavengers of superoxide anions. *Biochemical Pharmacology*.

[45] Seth P, Sundar SV, Seth RK (2003). Picroliv modulates antioxidant status and down-regulates AP1 transcription factor after hemorrhage and resuscitation. *Shock*.

[46] Gaddipati JP, Madhavan S, Sidhu GS, Singh AK, Seth P, Maheshwari RK (1999). Picroliv—a natural product protects cells and regulates the gene expression during hypoxia/reoxygenation. *Molecular and Cellular Biochemistry*.

[47] Anand P, Kunnumakkara AB, Harikumar KB, Kwang SA, Badmaev V, Aggarwal BB (2008). Modification of cysteine residue in p65 subunit of nuclear factor-*κ*B (NF-*κ*B) by picroliv suppresses NF-*κ*B-regulated gene products and potentiates apoptosis. *Cancer Research*.

[48] Mittal N, Gupta N, Saksena S, Goyal N, Roy U, Rastogi AK (1998). Protective effect of Picroliv from *Picrorhiza kurroa* against *Leishmania donovani* infections in *Mesocricetus auratus*. *Life Sciences*.

[49] Murthy SNS, Cooper HS, Shim H, Shah RS, Ibrahim SA, Sedergran DJ (1993). Treatment of dextran sulfate sodium-induced murine colitis by intracolonic cyclosporin. *Digestive Diseases and Sciences*.

[50] Ten Hove T, Van den Blink B, Pronk I, Drillenburg P, Peppelenbosch MP, Van Deventer SJH (2002). Dichotomal role of inhibition of p38 MAPK with SB 203580 in experimental colitis. *Gut*.

[51] Martín AR, Villegas I, Sánchez-Hidalgo M, De La Lastra CA (2006). The effects of resveratrol, a phytoalexin derived from red wines, on chronic inflammation induced in an experimentally induced colitis model. *British Journal of Pharmacology*.

[52] Kurtovic J, Segal I (2004). Recent advances in biological therapy for inflammatory bowel disease. *Tropical Gastroenterology*.

[53] Thukral C, Cheifetz A, Peppercorn MA (2006). Anti-tumour necrosis factor therapy for ulcerative colitis: evidence to date. *Drugs*.

[54] Aberra FN, Lichtenstein GR (2006). Infliximab in ulcerative colitis. *Gastroenterology Clinics of North America*.

[55] Feagan BG, Reinisch W, Rutgeerts P (2007). The effects of infliximab therapy on health-related quality of life in ulcerative colitis patients. *American Journal of Gastroenterology*.

[56] Zhang DK, Cheng LN, Huang XL, Shi W, Xiang JY, Gan HT (2009). Tetrandrine ameliorates dextran-sulfate-sodium-induced colitis in mice through inhibition of nuclear factor-*κ*B activation. *International Journal of Colorectal Disease*.

[57] Ishihara T, Tanaka KI, Tasaka Y (2009). Therapeutic effect of lecithinized superoxide dismutase against colitis. *Journal of Pharmacology and Experimental Therapeutics*.

[58] Zhou YH, Yu JP, Liu YF (2006). Effects of Ginkgo biloba extract on inflammatory mediators (SOD, MDA, TNF-*α*, NF-*κ*Bp65, IL-6) in TNBS-induced colitis in rats. *Mediators of Inflammation*.

[59] Liu XC, Mei Q, Xu JM, Hu J (2009). Balsalazine decreases intestinal mucosal permeability of dextran sulfate sodium-induced colitis in mice. *Acta Pharmacologica Sinica*.

[60] Kurutas EB, Cetinkaya A, Bulbuloglu E, Kantarceken B (2005). Effects of antioxidant therapy on leukocyte myeloperoxidase and Cu/Zn-superoxide dismutase and plasma malondialdehyde levels in experimental colitis. *Mediators of Inflammation*.

[61] Liu LN, Mei QB, Liu L (2005). Protective effects of *Rheum tanguticum* polysaccharide against hydrogen peroxide-induced intestinal epithelial cell injury. *World Journal of Gastroenterology*.

[62] Fridovich I, Pryor WA (1976). Oxygen radicals, hydrogen peroxide,and oxygen toxicity. *Free Radicals in Biology*.

[63] Suzuki Y, Matsumoto T, Okamoto S, Hibi T (2008). A lecithinized superoxide dismutase (PC-SOD) improves ulcerative colitis. *Colorectal Disease*.

[64] Uchimura K, Nagasaka A, Hayashi R (1999). Changes in superoxide dismutase activities and concentrations and myeloperoxidase activities in leukocytes from patients with diabetes mellitus. *Journal of Diabetes and its Complications*.

